# Erythrokeratodermia Variabilis-like Phenotype in Patients Carrying *ABCA12* Mutations

**DOI:** 10.3390/genes15030288

**Published:** 2024-02-24

**Authors:** Alrun Hotz, Regina Fölster-Holst, Vinzenz Oji, Emmanuelle Bourrat, Jorge Frank, Slaheddine Marrakchi, Mariem Ennouri, Lotta Wankner, Katalin Komlosi, Svenja Alter, Judith Fischer

**Affiliations:** 1 European Reference Networks (ERN Skin), 75015 Paris, France; alrun.hotz@uniklinik-freiburg.de (A.H.); vinzenz.oji@ukmuenster.de (V.O.); katalin.komlosi@uniklinik-freiburg.de (K.K.); svenja.alter@uniklinik-freiburg.de (S.A.); 2 Center for Cornification Disorders, Freiburg Center for Rare Diseases, Medical Center, University of Freiburg, 79106 Freiburg, Germany; 3 Institute of Human Genetics, Medical Center—University of Freiburg, Faculty of Medicine, University of Freiburg, 79106 Freiburg, Germany; 4 Department of Dermatology, Venerology and Allergology, University Medical Center Schleswig-Holstein, 24105 Kiel, Germany; rfoelsterholst@dermatology.uni-kiel.de; 5 Department of Dermatology and Venereology, Muenster University Medical Center, 48149 Muenster, Germany; 6 Department of Dermatology, Reference Center for Rare Skin Diseases MAGEC, Saint Louis Hospital AP-HP, 75015 Paris, France; emmanuelle.bourrat@aphp.fr; 7 Department of Dermatology, Venereology and Allergology, University Medical Center Göttingen, 37075 Göttingen, Germany; jorge.frank@med.uni-goettingen.de; 8 Department of Dermatology, CHU Hedi Chaker, Sfax University, Sfax 3029, Tunisia; slaheddine.marrakchi@tunet.tn; 9 Laboratory of Molecular and Functional Genetics, Faculty of Sciences of Sfax, Sfax University, Sfax 3029, Tunisia; mariamnouri1992@gmail.com

**Keywords:** erythrokeratodermia variabilis, autosomal recessive congenital ichthyosis (ARCI), *ABCA12*

## Abstract

Erythrokeratodermia variabilis (EKV) is a rare genodermatosis characterized by well-demarcated erythematous patches and hyperkeratotic plaques. EKV is most often transmitted in an autosomal dominant manner. Until recently, only mutations in connexins such as *GJB3* (connexin 31), *GJB4* (connexin 30.3), and occasionally *GJA1* (connexin 43) were known to cause EKV. In recent years, mutations in other genes have been described as rare causes of EKV, including the genes *KDSR*, *KRT83*, and *TRPM4*. Features of the EKV phenotype can also appear with other genodermatoses: for example, in Netherton syndrome, which hampers correct diagnosis. However, in autosomal recessive congenital ichthyosis (ARCI), an EKV phenotype has rarely been described. Here, we report on seven patients who clinically show a clear EKV phenotype, but in whom molecular genetic analysis revealed biallelic mutations in *ABCA12*, which is why the patients are classified in the ARCI group. Our study indicates that ARCI should be considered as a differential diagnosis in EKV.

## 1. Introduction

The term erythrokeratodermia describes a group of inherited skin disorders characterized by well-demarcated erythematous patches and hyperkeratotic plaques. These typical features may occur individually or in combination in affected individuals. Two major subtypes of erythrokeratodermia can be distinguished. The main subtype is classical erythrokeratodermia variabilis (EKV), formerly known as Mendes da Costa syndrome, which was initially described by da Costa [1]. The second and less common subtype is the progressive symmetric erythrokeratodermia (PSEK) [2]. Furthermore, there are atypical forms of erythroderatodermia, such as phenotypes resembling erythema gyratum repens [3].

In EKV, the erythematous patches are transient and migratory and can vary in size, shape, and number over a period of mostly days, but sometimes hours. These patches often show a map-like or annular morphology. The lesions usually occur in the first year of life and less frequently at birth or later in childhood or in early adult life [4]. The hyperkeratotic plaques are generally stable and localized or generalized. Depending on the severity, the hyperkeratoses can be reddish, yellow, or brown, up to a hystrix-like appearance [4]. EKV lesions predominantly appear on the limbs, buttocks, and lateral trunk in symmetric distributions. The face, scalp, and flexures are mostly spared. In some cases, palmoplantar hyperkeratosis occurs [4]. The hair, teeth, and nails are usually unaffected in EKV. After puberty, the lesions tend to stabilize or resolve spontaneously.

PSEK was originally designated as a distinct entity, but has overlapping features with EKV. The lesions in PSEK are nonmigratory, well demarcated, polycyclic, map-like-shaped, erythematous, and hyperkeratotic. They have a symmetrical distribution similar to EKV, but the trunk is typically spared [5]. However, due to the overlapping phenotypes and the similar genetic background of EKV and PSEK, the separate entity of PSEK has proven controversial to discuss. Some authors suggest the term erythrokeratodermia variabilis et progressiva (EKVP) to encompass both phenotypes [6].

EKV is predominantly transmitted in an autosomal dominant manner, with a high penetrance and considerable intra- and interfamilial variability. In rare cases, autosomal recessive inheritance caused by mutations in *GJB3* has been described [7,8]. In PSEK, autosomal recessive inheritance has been described more frequently. Heterozygous mutations for EKV have been detected in the genes *GJB3*, *GJB4*, and occasionally *GJA1*, encoding connexins 31, 30.3, and 43, respectively [9]. Connexines are a component of gap junctions, which provide channels for cell–cell communication. Gap junctions occur in almost all tissues, including the skin. In PSEK, mutations in *KDSR* [10], *KRT83* [11], and *TRPM4* [12] have been described as additional causative genes. Mutations in *KDSR* and *KRT83* have been described in autosomal recessive forms of PSEK, whereas mutations in *TRPM4* are inherited in an autosomal dominant manner.

The differential diagnosis of EKV mainly includes Netherton syndrome, which demonstrates migratory and serpiginous red plaques; however, there is a characteristic double-edged scaling in patients with ichthyosis linearis circumflexa, and, furthermore, psoriasis and epidermolytic ichthyosis. Autosomal recessive congenital ichthyosis (ARCI) is not a common differential diagnosis for EKV, as patients with ARCI usually do not show the characteristic well-demarcated erythematous patches. In ARCI, three major phenotypes have been described: lamellar ichthyosis (LI), congenital ichthyosiform erythroderma (CIE), and harlequin ichthyosis (HI). EKV-like phenotypes have only been described in isolated cases with ARCI, including patients with mutations in *NIPAL4* [13] and *ABCA12* [14]. Here, we present a larger cohort, including seven patients with initially suspected EKV, in which we found biallelic mutations in *ABCA12*, including four novel mutations and other known mutations previously described in patients with ARCI. The pathomechanism that leads to an EKV phenotype is not yet known. Our results indicate that an EKV phenotype is not uncommon in patients with *ABCA12* mutations and, therefore, ARCI should be considered for differential diagnosis in patients presenting an EKV phenotype.

## 2. Methods

Seven patients with suspected EKV were analyzed with NGS gene panels, including the classical EKV genes *GJB3*, *GJB4*, and *GJA1*, but also other causative genes for cornification disorders, such as ARCI.

Genomic DNA was isolated from peripheral blood lymphocytes. Subsequently, NGS methods were employed through a targeted multi-gene panel using HaloPlex Custom Kit or SureSelect Custom Kit (Agilent Technologies, Inc., Santa Clara, CA, USA). Resulting data were analyzed using an in-house bioinformatics pipeline and the commercial software SeqNext version 5.2.0 build 502 (JSI medical systems, Ettenheim, Germany).

Alignments were retrieved from Ensembl 109 [15] using Eutheria Gen Tree node. Analysis and visualization were performed with Jalview version 2.11.1.3-j1.8 [16]. The Genome Aggregation Database version v2.1.1 [17] and the ClinVar version September 2023 [18] were used. The classification of the detected sequence variants is based on the ACMG standards and guidelines [19].

## 3. Results

In all seven patients, P1–P7, in our study, the main suspected diagnosis was EKV. Mutations in the genes *GJB3*, *GJB4*, and *GJA1* were excluded in all patients. Detailed information about sex, origin, and detected mutations for all patients is summarized in Table 1. The mutations are biallelic in the patients.

**Figure 1:**
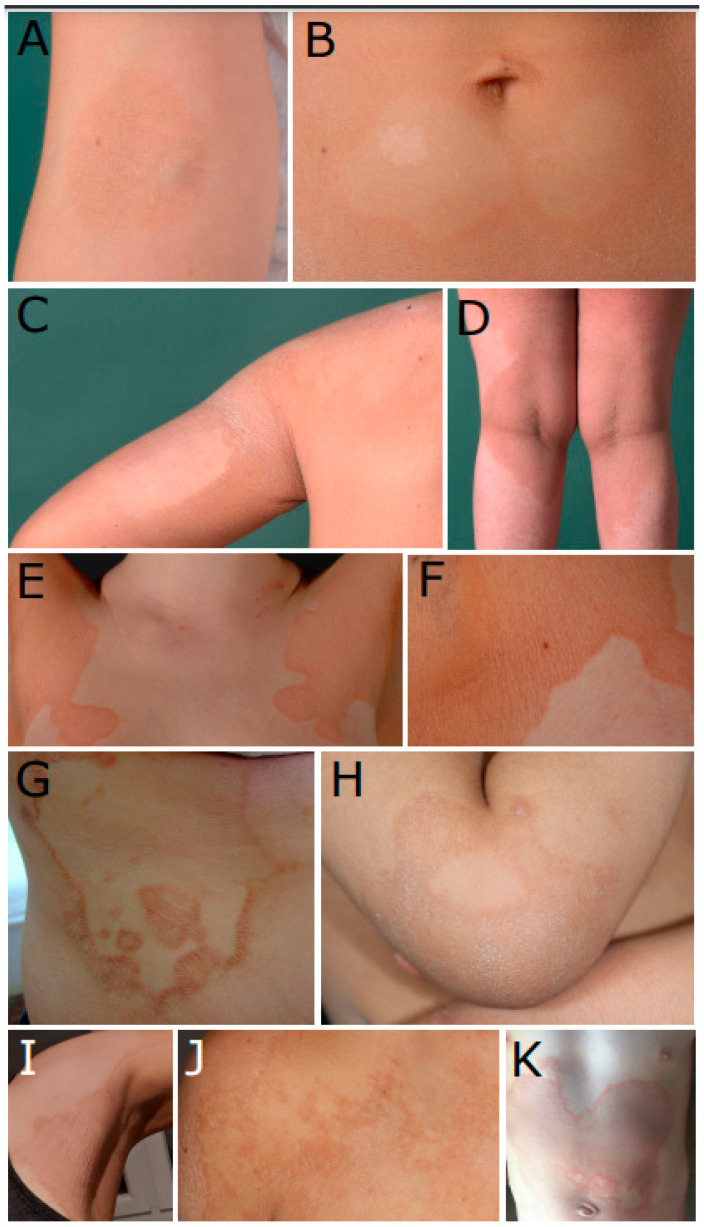
(**A**) Insular hyperkeratosis at the inside of the elbow and (**B**) erythrokeratodermic skin with fine scales at the abdomen, with sharply defined round areas with healthy skin and a hypopigmented spot in P1. (**C**) The shoulder and upper arm of P2 showing an erythrokeratoderma-like appearance, with moderate ichthyosis and inflammatory accentuated marginal areas and recessed areas in between. (**D**) Sharply demarcated erythrokeratodermic skin at the back of the thighs and knees in P2. (**E**,**F**) Sharply demarcated salmon-colored erythrokeratodermic plaques on the chest in P3. (**G**) Polycyclic erythematous and squamous plaques with red borders with fine scales on the trunk of P4. (**H**) Sharply demarcated erythrokeratodermic skin at the elbow of the daughter of P4. (**I**) Erythrokeratodermic plaques with fine white scales and dark reddened skin borders at the axilla, chest, and upper arm in P5; (**J**) sharply demarcated erythrodermic and hyperkeratotic skin with fine white scales in P6; (**K**) large erythrokeratodermic patches and a reddened skin border on the abdomen in P7.

P1 presented mild erythrokeratodermia since birth, with mild non-epidermolytic transgradient PPK. Insular hyperkeratotic lesions were found on the neck and abdomen and the skin folds of elbows and knees (Figure 1A,B). The patient was unable to sweat in the affected skin regions. Treatment with oral retinoids resulted in an improved skin appearance. Molecular genetic analysis revealed two heterozygous mutations in *ABCA12* (transcript ENST00000272895.7, NCBI reference sequence NM_173076.2, GRCh37.p13), including the splice-site mutation c.6962+1G>A, p.?. The mutation on the other allele, c.4139A>G, p.(Asn1380Ser), is the most frequent mutation in *ABCA12* [20]. Her brother, P2, was similar affected since birth. He presented erythrokeratodermia with moderate ichthyosis and inflammatory accentuated marginal areas, with recessed areas in between (Figure 1C,D). In contrast to his sister, P2 was first suspected to have a *PNPLA1*-associated ARCI. In P2, we detected the same mutations in *ABCA12* as in his sister.

In P3, the suspected diagnosis was EKV with skin abnormalities present since birth. The diagnosis of EKV was made at the age of about 2 years. Histologically, there was evidence for EKV or PRP. Between the ages of 17 and 23, she had almost no symptoms. Thereafter, salmon-colored, very-sharply demarcated plaques appeared again, which were prominent on the extremities but also on the face and chest. The plaques show fine scaling, especially at the edges (Figure 1E,F). Therapeutically, salicyl vaselin, glucocorticosteroids, UV therapy, and retinoids were applied. Two heterozygous mutations in *ABCA12* were found, c.130C>T, p.(Arg44Trp) and c.4544G>A, p.(Arg1515Gln). Both mutations have already been described in patients with ARCI.

P4 showed migrating polycyclic erythematous and squamous lesions on the trunk and limbs. The initial diagnosis was EKV; differentially, Netherton syndrome was suspected (Figure 1G). Netherton syndrome manifests in the skin through ichthyosis linearis circumflexa, which is marked by migratory erythematous plaques with a double-edged scale. This can show phenotypic similarities to EKV. We detected two homozygous variants each in *ABCA12*: c.6852G>C, p.(Glu2284Asp), which has already been described in “patients with lamellar ichthyosis”, and the variant c.3809A>G, p.(Tyr1270Cys), which is located in transmembrane domain 1. This patient, of Tunisian origin, was born to consanguineous parents, and her case has already been published by Ennouri et al. [21]. The phenotype was described there as ichthyosis linearis circumflexa. Her daughter was similarly affected (Figure 1H). Unfortunately, the DNA of her daughter was not available for molecular genetic analysis. Three siblings of P4 and her mother were also affected, and multiple consanguinity in the family was present, leading to a pedigree with pseudodominant inheritance.

P5 was initially diagnosed with Erythrokeratoderma progressiva et symmetrica. The symptoms started from six months of age. P5 presented well-demarcated erythematous patches and whitish hyperkeratotic plaques, especially on her arms and legs (Figure 1I). Two novel heterozygous mutations in *ABCA12* were detected: the nonsense mutation c.1270G>T, p.(Glu424*) and the missense mutation c.6611G>A, p.(Arg2204Gln), which is located in transmembrane domain 2.

P6 was diagnosed with EKV; her sister was also affected. P6 showed well-demarcated erythematous patches and partially hyperkeratotic plaques and mild palmoplantar hyperkeratosis (Figure 1J). Two novel compound heterozygous splice-site mutations in *ABCA12* were detected: c.2864-6T>A, p.? and c.2864-2A>T, p.? For both variants, prediction tools predict an impairment to the splicing process at the original splice site (Supplementary Materials Table S1).

P7 presented well-demarcated erythematous patches and hyperkeratotic plaques on his arms, legs, and trunk, mild palmoplantar hyperkeratosis, and dry and scaly skin (Figure 1K). Mutation analysis revealed two heterozygous mutations in *ABCA12*: the known nonsense mutation c.596G>A, p.(Trp199*) and the novel missense mutation c.6611G>A, p.(Arg2204Gln), which is located in transmembrane domain 2. Interestingly, the same missense mutation was found in P5. Both patients were of Caucasian origin, so a distant relationship cannot be ruled out.

## 4. Discussion

In this study, we present seven patients with a clinical diagnosis of EKV. All patients carry biallelic mutations in *ABCA12*. Biallelic pathogenic variants in *ABCA12* are usually described in ARCI. EKV is characterized by well-demarcated erythematous patches and hyperkeratotic plaques, whereas ARCI manifests in the three major phenotypes: lamellar ichthyosis, congenital ichthyosiform erythroderma, and the most severe form, harlequin ichthyosis. In ARCI, generalized ichthyosis is common, and well-demarcated erythematous patches are not characteristic.

An EKV-like phenotype in patients with mutations in genodermatoses-related genes has already been described in several studies. Two sisters with an EKV-like phenotype from a Tunisian consanguineous family carry a homozygous *NIPAL4* mutation [13]. The first symptoms in the older sister in this study appeared at the age of 5 months. In P4 in our study, whose case was initially published by Ennouri et al. [21], the symptoms also started from six months of age. ARCI is a condition in which the first symptoms are present at birth. Since a later onset of symptoms is atypical for ARCI, this often leads to misdiagnosis. The younger sister in the study by Charfeddine et al. [13] exhibited an ichthyosiform-like appearance, suggesting the ARCI condition. This is similar to P2, who was first suspected to have ARCI, whereas his sister presents a more classical EKV phenotype. These examples show that there can be large phenotypic differences within the family, which may lead to misdiagnosis and highlight the need for molecular genetic testing.

An EKV-like phenotype has also been reported in other genodermatoses besides ARCI. Pujol et al. [22] reported a 4-year-old boy presenting generalized ichthyosiform skin manifested by migrating scaly plaques alternating with areas of normal-looking skin, showing erythematous borders with sharp margins. The detection of biallelic mutations in *ABHD5* led to the diagnosis of Chanarin–Dorfman syndrome [22]. Biallelic *ELOVL4* mutations lead to ichthyosis, spastic quadriplegia, and impaired intellectual development. Cadieux-Dion et al. [23] demonstrate a patient with biallelic *ELOVL4* mutations presenting an EKV-like skin phenotype compared with other patients with the same gene defect who present with ichthyosis.

Different phenotypes in patients with mutations in the same gene may depend on several genetic factors: the type of mutation, the location of the mutation in specific domains, and the specific mutation itself. In the *ABCA12* gene, the type of mutation determines the phenotype: truncating variants lead to harlequin ichthyosis, whereas missense variants lead to congenital ichthyosiform erythroderma or lamellar ichthyosis. For missense mutations, the location within the protein is important. Pathogenic missense mutations are located primarily in transmembrane and ATP-binding cassette domains [20,24]. Mutation-specific phenotypes have been described, for example, in the *TGM1* gene: Raghunath et al. [25] described a particular mutation leading to a self-healing collodion baby. Oji et al. [26] found that particular mutations affect the TGase-1 function depending on temperature, which results in bathing suit ichthyosis. In our cohort of patients with *ABCA12* mutations, we did not find any genotype–phenotype correlation. Our patients carry truncating and missense variations as well as splice-site mutations, so the type of mutation does not appear to determine the EKV phenotype. Furthermore, some mutations were described in different phenotypes such as ARCI and EKV in different families and even within a family. Interestingly, the novel missense mutation c.6611G>A, p.(Arg2204Gln) was found in two patients in our cohort. It cannot be excluded that specific *ABCA12* mutations can potentially lead to an EKV phenotype. Recently, Terrinoni et al. [14] described two patients from a family showing an EKVP phenotype who carry two missense mutations in *ABCA12*. The authors speculated that the detected mutations c.4412A>G, p.(His1471Arg) and c.4601C>T, p.(Thr1534Met) do not completely abolish *ABCA12* activity, which could lead to an intermediate phenotype resembling EKVP. However, both mutations have already been described in two patients with ARCI in Hotz et al. [20]: the mutation c.4412A>G was detected compound heterozygous with a second pathogenic variant in patient P34; the variant c.4601C>T was found in a homozygous state in patient P5 in this publication. Neither patient showed any features of EKV. This indicates that the existence of EKV-specific mutations cannot be confirmed at this time. It is possible that further genetic, multifactorial, or environmental factors contribute to an EKV phenotype.

Of particular interest in our cohort is patient 4, in whom two homozygous variants were detected in *ABCA12*: the variants c.6852G>C, p.(Glu2284Asp) and c.3809A>G, p.(Tyr1270Cys). The first variant is located in ATP-binding cassette 2, whereas the second variant is located in transmembrane domain 1. Most missense mutations are located in transmembrane domains or ATP binding cassettes [20]; therefore, a pathogenicity of both variants is probable. Unfortunately, the other affected family members are not available for analysis. However, it can be assumed that each allele carries both variants c.6852G>C and c.3809A>G. An additional influence of a second missense variant in cis on the protein function is generally possible. Since P4 is similarly affected, like the other patients in the cohort, there is no evidence that the presence of two missense mutations exacerbates the phenotype.

EKV is a phenotype that does not normally occur in patients with ARCI. This has so far only been described in isolated cases with ARCI and other genodermatoses. Our cohort shows that an EKV phenotype in ARCI patients is not just an exceptional case, but more common than expected. Our study aims to sensitize diagnosticians to consider other differential diagnoses in the presence of an EKV phenotype. In patients with an EKV phenotype, panel diagnostics should be performed that include ARCI genes and other genodermatose genes in addition to classical EKV genes.

## Figures and Tables

**Table 1:** Patients with the EKV phenotype carrying mutations in *ABCA12* (novel mutations are in bold).

Patient	Sex	Age	Origin	Mutation 1	Mutation 2
1	f	35 y	Caucasian	c.4139A>G, p.(Asn1380Ser)	c.6962+1G>A, p.?
2	m	28 y	Caucasian	c.4139A>G, p.(Asn1380Ser)	c.6962+1G>A, p.?
3	f	29 y	Caucasian	c.130C>T, p.(Arg44Trp)	c.4544G>A, p.(Arg1515Gln)
4	f	38 y	North African	c.3809A>G, p.(Tyr1270Cys)homozygous	c.6852G>C, p.(Glu2284Asp)homozygous
5	f	25 y	Caucasian	c.1270G>T, p.(Glu424*)	**c.6611G>A, p.(Arg2204Gln)**
6	f	33 y	North African	**c.2864-6T>A, p.?**	**c.2864-2A>T, p.?**
7	m	9 y	Caucasian	c.596G>A, p.(Trp199*)	**c.6611G>A, p.(Arg2204Gln)**

## Data Availability

Data are contained within the article and Supplementary Materials.

## Supplementary Materials

The following supporting information can be downloaded at: https://www.mdpi.com/article/10.3390/genes15030288/s1, Table S1: Classification of the detected variants in *ABCA12* [27,28,29,30,31].
