# Supplementary material for: Erythrokeratodermia Variabilis-like Phenotype in Patients Carrying ABCA12 Mutations
Source: Genes (Basel). 2024 Feb 24;15(3):288. doi: 10.3390/genes15030288 (PMC10970099; doi:10.3390/genes15030288)
Supplement: Supplementary file 1 [file genes-15-00288-s001.zip › genes-2857160-supplementary.pdf]

Supp. Table S1: classification of the detected variants in *ABCA12*

Mutation		gnomAD	ClinVar	Splice prediction tools	ACMG classification	literature
c.130C>T	p.(Arg44Trp)	0x homo<br>8x het<br>MAF 0.003%	2x likely pathogenic	-	pathogenic (PM2, PM5, PP3, PP5)	Scott *et al*. (2013)
c.596G>A	p.(Trp199*)	n/a	1x pathogenic	-	pathogenic (PVS1, PM2, PM3, PP5)	Thomas *et al*. (2006)
**c.1270G>T**	**p.(Glu424*)**	n/a	n/a	-	pathogenic (PVS1, PM2)	**this study**
**c.2864-6T>A, p.?**	**p.?**	0x homo<br>16x het<br>MAF 0.006%	n/a	reduction of score of wt acceptor splice site from 0.90 to 0.42 (NN) and from 0.63 to 0.23 (NG2)	uncertain significance (PM2, PM3)	**this study**
**c.2864-2A>T, p.?**	**p.?**	n/a	n/a	NN, NG2: loss of wt acceptor splice site	pathogenic (PVS1, PM2)	**this study**
c.3809A>G	p.(Tyr1270Cys)	n/a	n/a	-	likely pathogenic (PM1, PM2, PM3, PP3)	Ennouri *et al*. (2022)
c.4139A>G	p.(Asn1380Ser)	0x homo<br>5x het<br>MAF 0.002%	2x likely pathogenic, 5x pathogenic	-	pathogenic (PM1, PM2, PP3, PP5)	Lefévre *et al*. (2003)
c.4544G>A	p.(Arg1515Gln)	0x homo<br>3x het<br>MAF 0.001%	n/a	-	pathogenic (PM1, PM2, PP3, PP5)	Israeli *et al.* (2013)
**c.6611G>A**	**p.(Arg2204Gln)**	0x homo<br>89x het<br>MAF 0.03%	1x likely benign, 1x uncertain significance	-	likely pathogenic (PM1, PM2, PM3, PP3)	**this study**
c.6852G>C	p.(Glu2284Asp)	0x homo<br>30x het<br>MAF 0.01%	n/a	-	pathogenic (PM1, PM2, PM3, PP3, PP5)	Bastaki *et al*. (2017), Ennouri *et al*. (2022)
c.6962+1G>A	p.?	0x homo<br>10x het<br>MAF 0.004%	n/a	NN, NG2: loss of wt donor splice site	pathogenic (PVS1, PM2, PP5)	Hou *et al*. (2020)

n/a: not listed in the database. Homo: homozygous. Het: heterozygous. MAF: minor allele frequency, NN: NNSplice. NG2: NetGene2. wt: wild-type. -: not evaluated.

gnomAD: The Genome Aggregation Database version v2.1.1 (http://gnomad.broadinstitute.org/). The data set provided on this website spans 125,748 exome sequences and 15,708 whole-genome sequences from unrelated individuals sequenced as part of various disease-specific and population genetic studies.
ClinVar: Version september 2023 (https://www.ncbi.nlm.nih.gov/clinvar/).

NNSplice: Splice site predictions for 1 sequence with donor score cutoff 0.40, acceptor score cutoff 0.40. Score ranging from 0 to 1, higher score implies a more potential splice site.
NetGene2: Cutoff values used for confidence: highly confident donor sites (H): 95.0 %, nearly all true donor sites: 50.0 %, highly confident acceptor sites (H): 95.0 %, nearly all true acceptor sites: 20.0 %. Confidence score ranging from 0 to 1, higher score implies a higher confidence of true site.
ACMG: American College of Medical Genetics and Genomics, Richards *et al*. (2015).

Scott *et al*. Targeted sequence capture and high-throughput sequencing in the molecular diagnosis of ichthyosis and other skin diseases. J Invest Dermatol. 2013 Feb;133(2):573-6.
Thomas *et al*. ABCA12 is the major harlequin ichthyosis gene. J Invest Dermatol. 2006 Nov;126(11):2408-13.
Ennouri *et al*. Clinical and genetic investigation of ichthyosis in familial and sporadic cases in south of Tunisia: genotype-phenotype correlation. BMC Med Genomics. 2022 Jan 5;15(1):4.
Lefévre *et al*. Mutations in the transporter ABCA12 are associated with lamellar ichthyosis type 2. Hum Mol Genet. 2003 Sep 15;12(18):2369-78.
Israeli *et al.* Non-syndromic autosomal recessive congenital ichthyosis in the Israeli population.
Bastaki *et al*. Summary of mutations underlying autosomal recessive congenital ichthyoses (ARCI) in Arabs with four novel mutations in ARCI-related genes from the United Arab Emirates. Int J Dermatol. 2017 May;56(5):514-523.
Hou *et al*. Precision medicine integrating whole-genome sequencing, comprehensive metabolomics, and advanced imaging. Proc Natl Acad Sci U S A. 2020 Feb 11;117(6):3053-3062.
